# Association between Tumor Necrosis Factor-α 308G/A Gene Polymorphism and Silicosis Susceptibility: A Meta-Analysis

**DOI:** 10.1371/journal.pone.0076614

**Published:** 2013-10-04

**Authors:** Zhanzhan Li, Jing Xue, Shipeng Yan, Peng Chen, Lizhang Chen

**Affiliations:** 1 Department of Epidemiology and Health Statistics, School of Public Health, Central South University, Changsha, Hunan Province, China; 2 Xiangya Medical School, Central South University, Changsha, Hunan Province, China; University of Texas Health Science Center at Houston, United States of America

## Abstract

**Background:**

Tumor necrosis factor-α (TNF-α) 308 G/A gene polymorphism has been reported to be associated with susceptibility to silicosis. However, the relevant study results are still inconsistent.

**Objective and Methods:**

A meta-analysis was performed in order to drive a more precise estimation of the relationship between TNF-α-308 G/A gene polymorphism and susceptibility to silicosis. Electronic databases were searched and nine separate studies were included. The pooled odds ratios (ORs) and the corresponding 95% confidence internal (CI) were calculated by a fixed effect model.

**Results:**

A total of 1267 cases and 1214 controls were included. In the overall analysis, significantly increased silicosis risk was found (for GA+AA vs. GG *OR*=1.45, 95%CI: 1.20-1.760, *P*=1.58E4; for GA vs. GG: OR=1.53, 95%CI=1.25-1.86, *P*=3.11E5; for A allele vs. G allele: OR=1.27, 95%CI=1.08-1.50, *P*= 0.004). In the subgroup analysis, significantly increased silicosis risk was also found among Asians (for GA+AA vs. GG: OR=1.63, 95%CI=1.27-2.08, *P*=1.01E4), for GA vs. GG: OR=1.71, 95%CI=1.33-2.20, *P*=3.44E5), for A allele vs. G allele: OR=1.45, 95%CI=1.17-1.80, *P*=0.001). However, no significantly increased risk was found among non-Asians for all genetic models.

**Conclusions:**

TNF-α-308 G/A polymorphism might lead to an increased risk of silicosis susceptibility, especially for Asians. However, further studies with large sample sizes should be conducted to confirm the association.

## Introduction

Silicosis is one of the most important occupational diseases worldwide [1], which is considered a major public health problem in some developing countries such as India, South African and China. China has the largest number of silicosis patients with 6000 new cases and more than 24000 deaths every year [2]. In the developed countries, although a steady decline in death rates had happened due to protective measures, new outbreaks still occur occasionally [3,4]. The risk of disease is mainly related to the amount of silica inhaled through a working life-time and once established there are still no effective treatment approaches at present [5]. Moreover, it is well established that patients with silicosis are at high risk of developing other diseases such as lung cancer and tuberculosis (TB) [6-10]. In a follow up study with an average duration of exposure to free silica of 16.8 years, an odds ratio (OR) of 2.75 was reported for the association of TB with silicosis [11]. However, the pathogeny of silicosis has not yet been fully illustrated. Current evidence from experimental and clinical studies proves that the pathologic process of silicosis is promoted by the increased secretion of some proinflammatory cytokines such as tumor necrosis factor-alpha (TNF-α) [12-14]. There is a wide inter-individual variability of susceptibility to silicosis as with other multifactor diseases [15]. Some studies show the individual variation in susceptibility to silicosis, which means that genetic factors may affect the susceptibility to this disease [16,17].

TNF-α is an important mediator of inflammatory response, and is involved in the pathogenesis of various human autoimmune and inflammatory diseases [18,19]. TNF-α gene is located on chromosome 6 (region p21.3), within the central major histocompatibility complex [20] and plays a key role in the regulation of fibrotic process by increasing the release of TNF-α from alveolar macrophages [21-23]. Several previous studies have also discovered that TNF-α production is regulated at the transcriptional level [24-26], and a G-to-A mutation in the -308 promoter section is accompanied by an increase in TNF-α production. There are already population-based studies on the association between silicosis risks and TNF-α gene polymorphism (308G/A, rs1800629) have been carried out. However, several results in different studies have been conflicting. It is verified that the OR of disease for carriers of the minor variant, TNF-α, is markedly higher for severe silicosis and significantly lower for moderate silicosis [27]. Regardless of disease severity, the ORs disease for carriers of the TNF-α-308G/A variants are elevated. A predominant effect on disease severity, rather than on disease frequency, appears to be a general feature of promoter polymorphism in diseases in which TNF- α has a critical role in black South African Miners [28]. On the contrary, study performed by Wu [29] did not find the association between TNF-α gene at position -308G/A and severity of silicosis, and genetic variants might not play a dominant role in the association with silicosis in the Chinese population. Cytokine polymorphisms of TNF-α were found to be associated with the silicosis risk in the Chinese workers exposed to silica particles [30]. Other several studies also have different conclusions [31-35].

In the current study, a meta-analysis of nine individual studies was conducted to determine whether there was a relationship between TNF-α-308G/A polymorphism and silicosis risks in the whole population.

## Materials and Methods

### Publication search

PubMed, Web of Science, Embase, China National Knowledge Infrastructure for relevant studies published in English or Chinese(CNKI), WanFang, Database of Chinese Scientific and Technical Periodicals (VIP) were systematically searched using the following search terms: “tumor necrosis factor-α” or “tumor necrosis factor-alpha” or “TNF-α” or “TNF-alpha” and “silicosis” combined with “polymorphism” or “mutation” or “variant ” and the last search was updated to April 25, 2013. Animal studies or non-English and Chinese language articles were not included. All objects included studies were approved by the Medical Ethics Committee.

### Inclusion criterion

The inclusion criteria are: 1) information on the evaluation of TNF-α gene-308G/A polymorphism and the susceptibility to silicosis; 2) sufficient published data for estimating the odds ratio (OR) with 95% confidence interval (CI); 3) studies using a case-control design; 4) not republished data; 5) not animal studies.

### Data extraction

Information was extracted from all selected publications by two investigators separately. If these two investigators could not reach a consensus; disagreements were discussed and resolved by a third investigator. For each of the included articles, information including first author, year of publication, source of controls, study population (ethnicity), total numbers of patients and controls, and frequencies of TNF-α gene-308G/A polymorphism in patients and controls was extracted.

### Statistical analysis

The association between TNF-α-308 G/A polymorphism and the risk of silicosis was estimated by calculating a pooled OR and 95%CI under a dominant model (GA+AA vs. GG), a recessive model (AA vs. GA+GG), additive model (AA vs. GG) and an allele model (A vs. G). The fixed-effects model or random-effects model was used depending on whether or not heterogeneity existed among studies. A subgroup analysis was carried out with respect to ethnicity. Heterogeneity among studies was examined with Cochran’s *Q* statistic and the *I* ^*2*^statistic. *P* <0.10 in *Q*-test indicated no heterogeneity among studies. *P*<0.1 rather than 0.05 was considered significant heterogeneity for the *χ* ^*2*^-based *Q* testing and a value of 0% for *I* ^2^ indicated no heterogeneity and increasing percentage implied increased heterogeneity. That is, *I* ^2^ will be used to estimate total variation across studies that are due to heterogeneity rather than chance (<25% is considered low heterogeneity, 25%–50% moderate, and >50% as high-level heterogeneity) [36,37]. The statistical significance of *OR* was analyzed using *Z* test, and *P* < 0.05 was considered as statistically significant. Sensitivity analysis was performed by sequentially excluding individual studies to assess the stability of the results [38]. Hardy-Weinberg equilibrium was assessed by the χ^2^ test in the controls [39]. The potential publication bias was examined by using the funnel plot [40]. Funnel plot asymmetry was assessed by the method of Egger’s linear regression test (*P*<0.05 was considered representative of statistically significant publication bias). Statistical analysis was performed with Stata11.0 (Stata Corporation, College Station, TX), and all *P*-values were two-tailed. Power analysis was performed by Power Analysis (PASS) 2008(http://www.ncss.com/pass.html)

## Results

### Studies and populations

**Figure 1:**
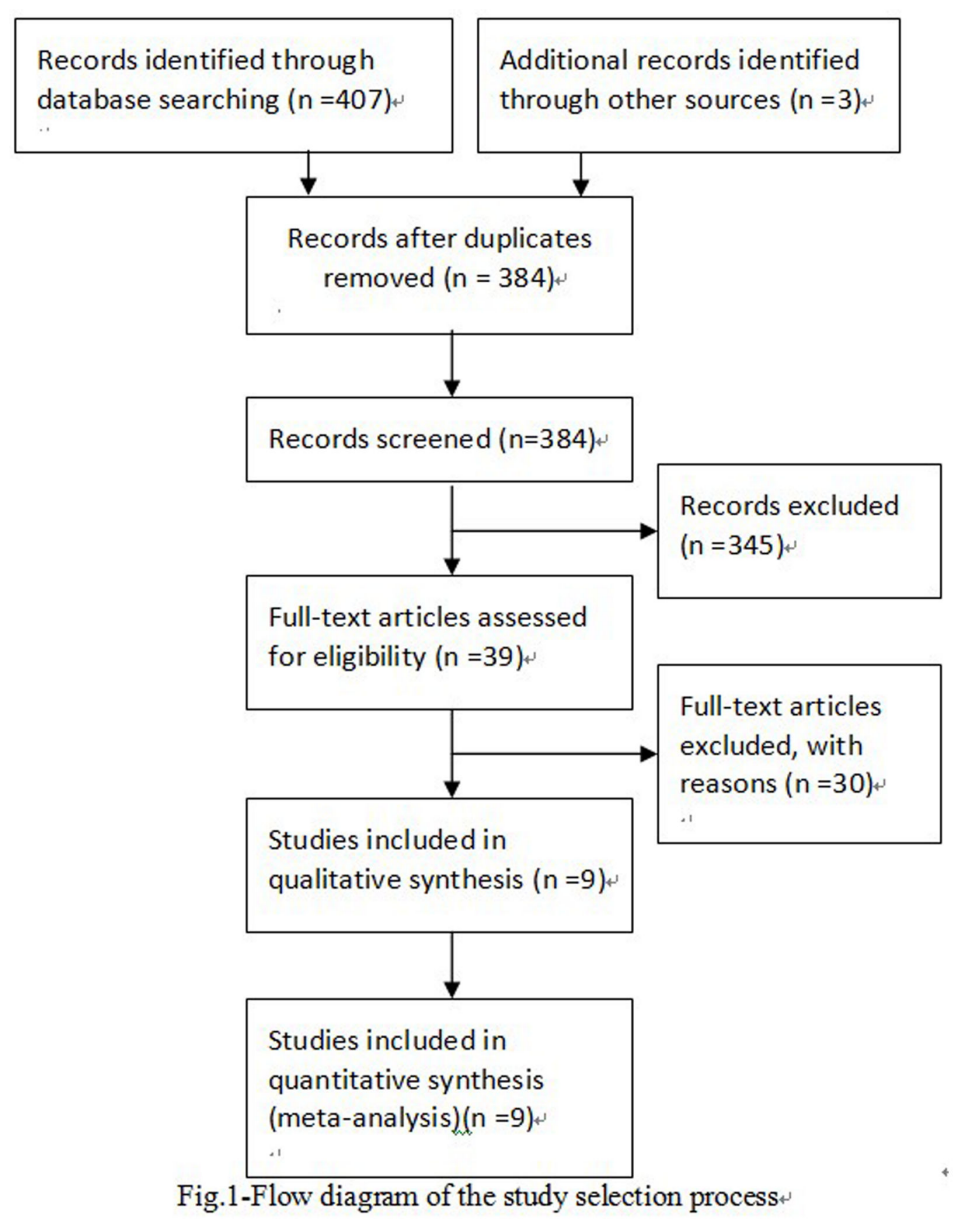
Flow diagram of included/excluded studies.

The flow diagram of the search process is exhibited in Figure 1

The initial search of databases identified 410 potentially relevant articles, of which 401 articles (including 81 animal studies, 282 not silicosis or TNF-α gene-308G/A researches) were rejected because of their obvious irrelevance to the purpose of this study. In addition, 9 reviews, 3 meeting abstracts and 26 republished studies were excluded. Finally, a total of nine published articles including 1267 cases and 1214 controls reported the relationship between TNF-α-308G/A gene polymorphism and the susceptibility to silicosis. The countries of these studies include China, USA, and South Africa. Seven studies involving Asians include 852 patients and 940 controls, and the two studies involving non-Asians include 415 patients and 274 controls. The total nine studies provide the numbers of allele A and allele G in both silicosis patients and controls. The Hardy–Weinberg equilibrium had been tested for all polymorphisms in the control groups. Table 1 and Table 2 present the main characteristics and data of these studies.

**Table 1:** Main data of studies included in the meta-analysis.

First author, year	Genotype distribution cases/control				
	GG	GA	AA	G	A
Wang,2005	0.60/0.77	0.36/0.16	0.04/0.07	0.78/0.85	0.22/0.15
Wu,2007	0.80/0.86	0.18/0.13	0.02/0.02	0.89/0.92	0.11/0.08
Yang,2005	0.66/0.76	0.34/0.21	0.00/0.03	0.83/0.86	0.17/0.14
Li,2004	0.86/0.88	0.13/0.11	0.01/0.01	0.93/0.94	0.07/0.06
Yucesoy,2001	0.42/0.49	0.56/0.49	0.02/0.03	0.70/0.73	0.30/0.27
Wang,2005	0.87/0.96	0.13/0.04	0.00/0.00	0.94/0.98	0.06/0.02
Wang,2012	0.62/0.76	0.340.22	0.04/0.01	0.79/0.88	0.21/0.13
Isa,2012	0.42/0.49	0.47/0.44	0.11/0.07	0.66/0.71	0.340.29
Corbett,2002	0.60/0.61	0.34/0.30	0.06/0.09	0.77/0.76	0.23/0.24

**Table 2:** Frequencies of genotype.

First author (year)	County	Source of control	Genotyping method	Simple size cases/control	Genotype distribution cases/control					HWE Y/N(P)
					GG	GA	AA	G	A	
Wang,2005	China	PB	PCR-RFLP	75/137	45/106	27/22	3/9	117/234	33/40	Y(0.149)
Wu,2007	China	PB	PCR-RFLP	183/111	147/95	33/14	3/2	327/204	39/18	Y(0.105)
Yang,2005	China	PB	PCR-RFLP	96/116	63/88	33/24	0/4	159/200	33/32	Y(0.162)
Li,2004	China	PB	PCR-RFLP	259/341	224/300	33/39	2/2	481/639	37/43	Y(0.555)
Yucesoy,2001	USA	PB	PCR-RFLP	294/154	123/75	166/75	5/4	412/225	176/83	N(0.003)
Wang,2005	China	PB	PCR-RFLP	126/122	110/117	16/5	0/0	236/239	16/5	Y(0.817)
Wang,2012	China	PB	PCR-RFLP	68/68	42/52	23/15	3/1	107/119	29/17	Y(0.944)
Isa,2012	Iran	PB	PCR-RFLP	45/45	19/22	21/20	5/3	59/64	31/26	Y(0.584)
Corbett,2002	South Africa	PB	PCR-RFLP	121/120	73/73	41/36	7/11	187/182	55/58	Y(0.054)

### Pooled analyses

**Figure 2:**
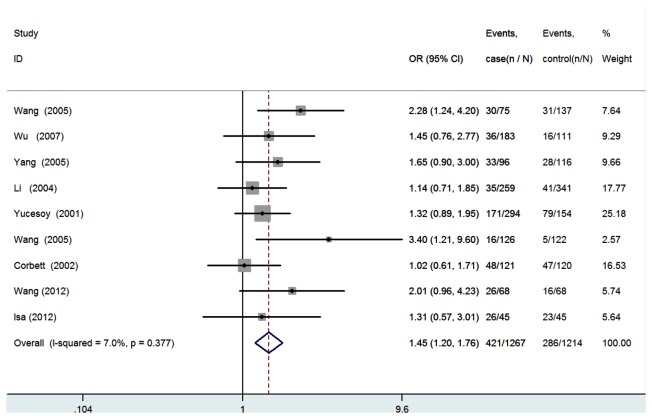
Forest plot of the susceptibility of silicosis associated with TNF-α-308 G/A gene ( **GA**/**AA** versus GG).

**Figure 3:**
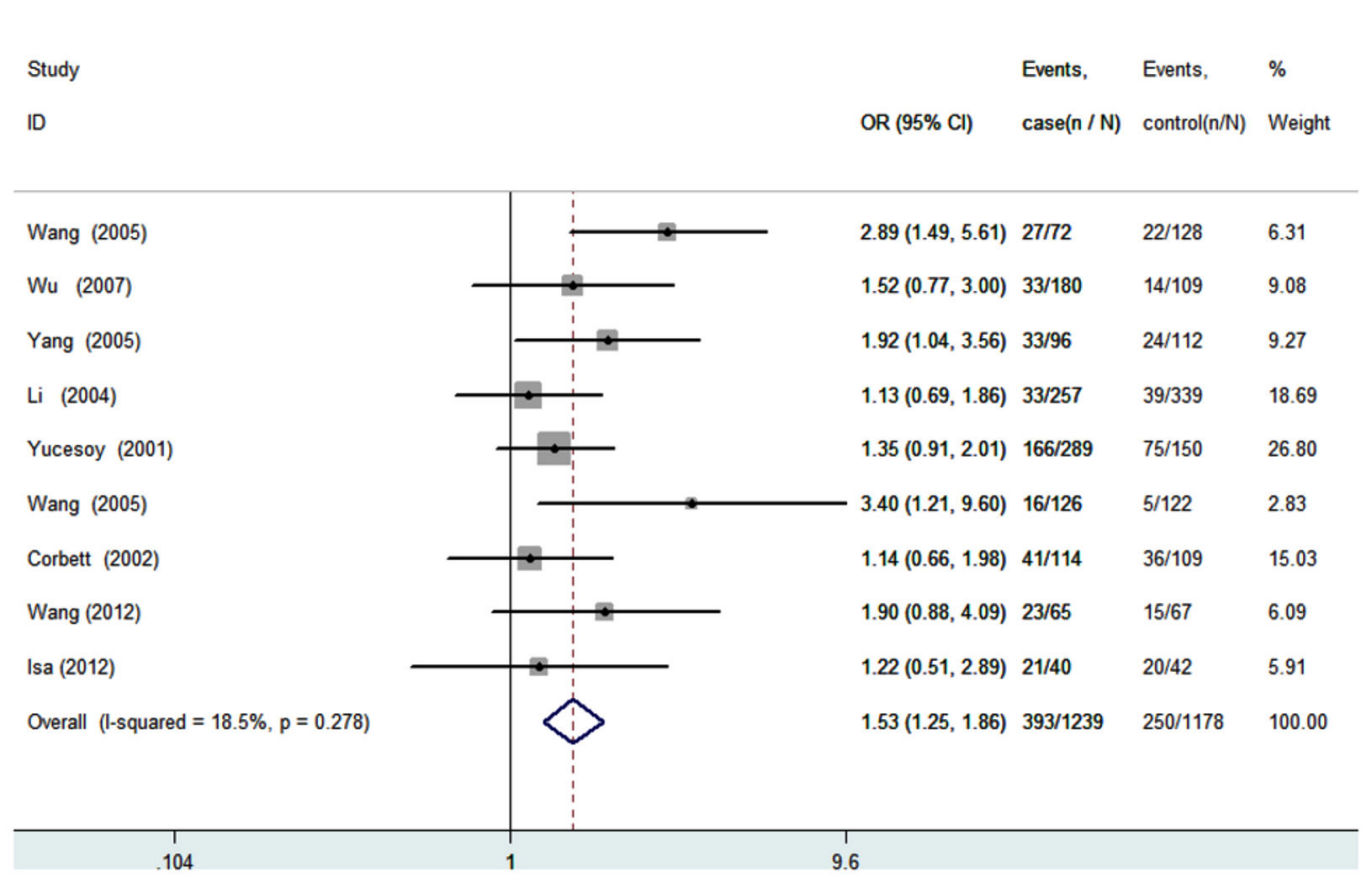
Forest plot of the susceptibility of silicosis associated with TNF-α-308 G/A(GA vs. GG).

**Figure 4:**
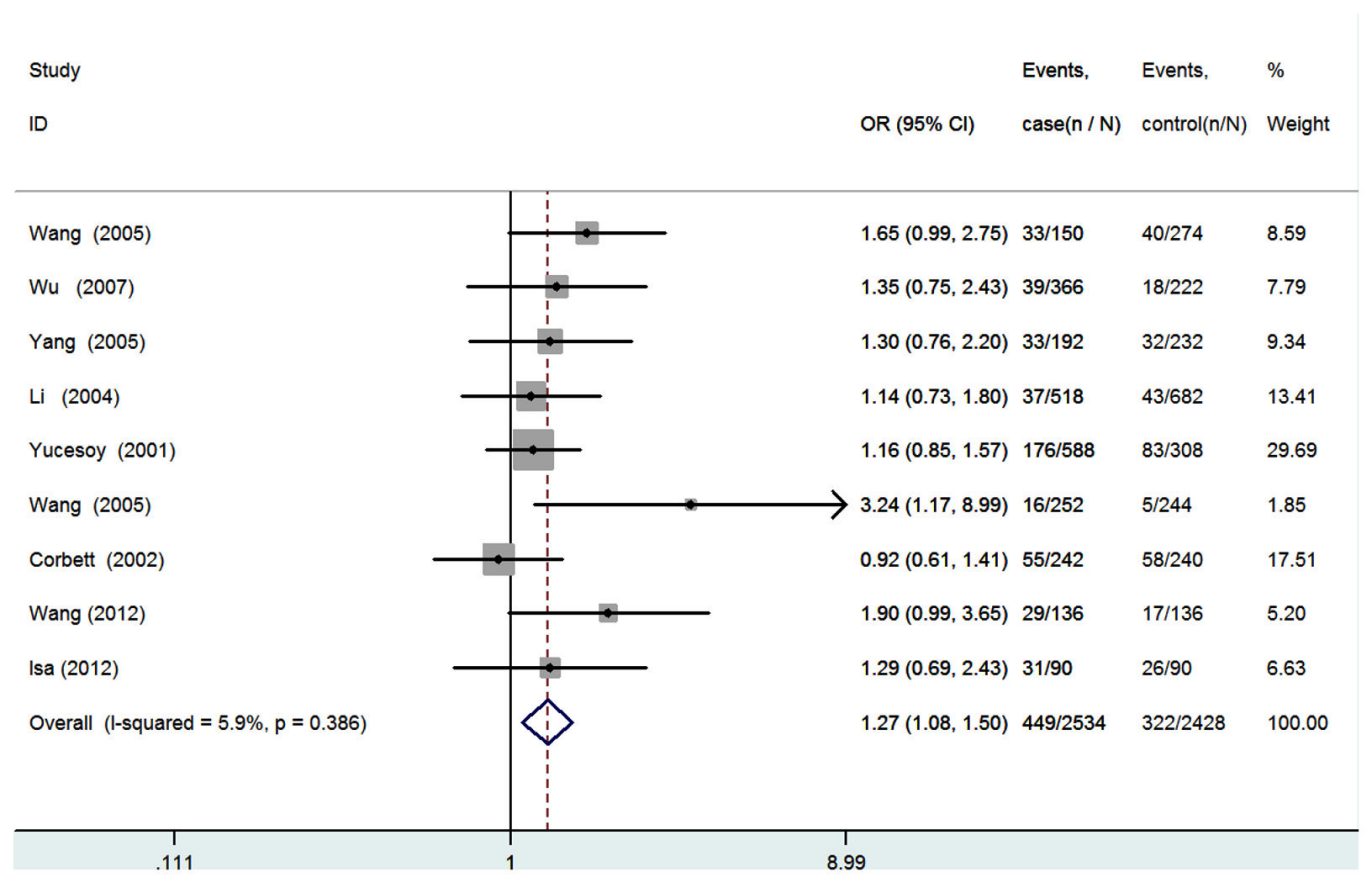
Forest plot of the susceptibility of silicosis associated with TNF-α308 G/A(A allele vs. G allele).

All nine studies were pooled into the meta-analysis. There was no evidence of between-study heterogeneity under the additive model (AA vs. GG: *I* ^2^=0.0%, *P* for heterogeneity =0.753; GA vs. GG: *I* ^2^=18.5%, *P*=0.278), the dominant model (GA+AA vs. GG: *I* ^2^=7.0%, *P*=0.377), the recessive model (AA vs. GA+GG: *I* ^2^=0.0%, *P* =0.692), and the allele model (A vs. G:I^*2*^=5.9%, *P* =0.386). Therefore, a fixed-effects model was used for all the genetic models. In overall analysis, significantly increased silicosis risk was found for GA+AA vs. GG (*OR*=1.45, 95%CI=1.20-1.76, *P*=1.58E4, Figure 2), for GA vs. GG (*OR*=1.53, 95%CI=1.25-1.86, *P*=3.11E5, Figure 3), and for A allele vs. G allele (*OR*=1.27, 95%CI=1.08-1.50, *P*= 0.004, Figure 4). The power analysis shows that our study has a power greater than 80% to detect the effects of TNF-α polymorphism on silicosis susceptibility, assuming an *OR* of 1.45.Then subgroup analysis was performed to analysis the potential ethnic differences. Only non-Asians and Asians were compared because only one study was carried out on Caucasians or Africans. The fixed-effects model was used for all the genetic models because of the absence of heterogeneity. Significantly increased silicosis risk was also found for GA+AA vs. GG (*OR*=1.63, 95%CI=1.27-2.08, *P*=1.01E4), for GA vs. GG (*OR*=1.71, 95%CI=1.33-2.20, *P*=3.44E5), and for A allele vs. G allele (*OR*=1.45, 95%CI=1.17-1.80, *P*= 0.001), but, not significantly increased risk was found amongnon-Asians for all genetic models (Table 3).

**Table 3:** Summary of different comparative results.

Category	Genetic model		*OR*(*95%CI*)	*Z*	*P* value	*I* ^2^%	*P*_het*_	Effect model
Overall	Dominant	AA+GA vs. GG	1.45[1.20,1.76]	3.78	1.58E4	7.0	0.377	Fixed
	Recessive	AA vs. GA+GG	0.77[0.46,1.29]	0.98	0.326	0.0	0.692	Fixed
	Additive	AA vs. GG	0.79[0.45,1.37]	0.84	0.398	0.0	0.753	Fixed
	Allele	A vs. G	1.27[1.08,1.50]	2.88	0.004	5.9	0.386	Fixed
Asian	Dominant	AA+GA vs. GG	1.63[1.27,2.08]	3.89	1.01E4	0.0	0.439	Fixed
	Recessive	AA vs. GA+GG	0.91[0.46,1.77]	0.29	0.772	0.0	0.540	Fixed
	Additive	AA vs. GG	0.90[0.42,1.96]	0.26	0.796	0.0	0.552	Fixed
	Allele	A vs. G	1.45[1.17,1.80]	3.36	0.001	0.0	0.585	Fixed
nonAsian	Dominant	AA+GA vs. GG	1.20[0.88,1.64]	1.15	0.249	0.0	0.438	Fixed
	Recessive	AA vs. GA+GG	0.62[0.28,1.37]	1.18	0.239	0.0	0.939	Fixed
	Additive	AA vs. GG	0.68[0.30,1.51]	0.95	0.341	0.0	0.833	Fixed
	Allele	A vs. G	1.07[0.84,1.37]	0.54	0.588	0.0	0.394	Fixed

*P*_het_=*P* value for heterogeneity

### Sensitivity analysis

**Figure 5:**
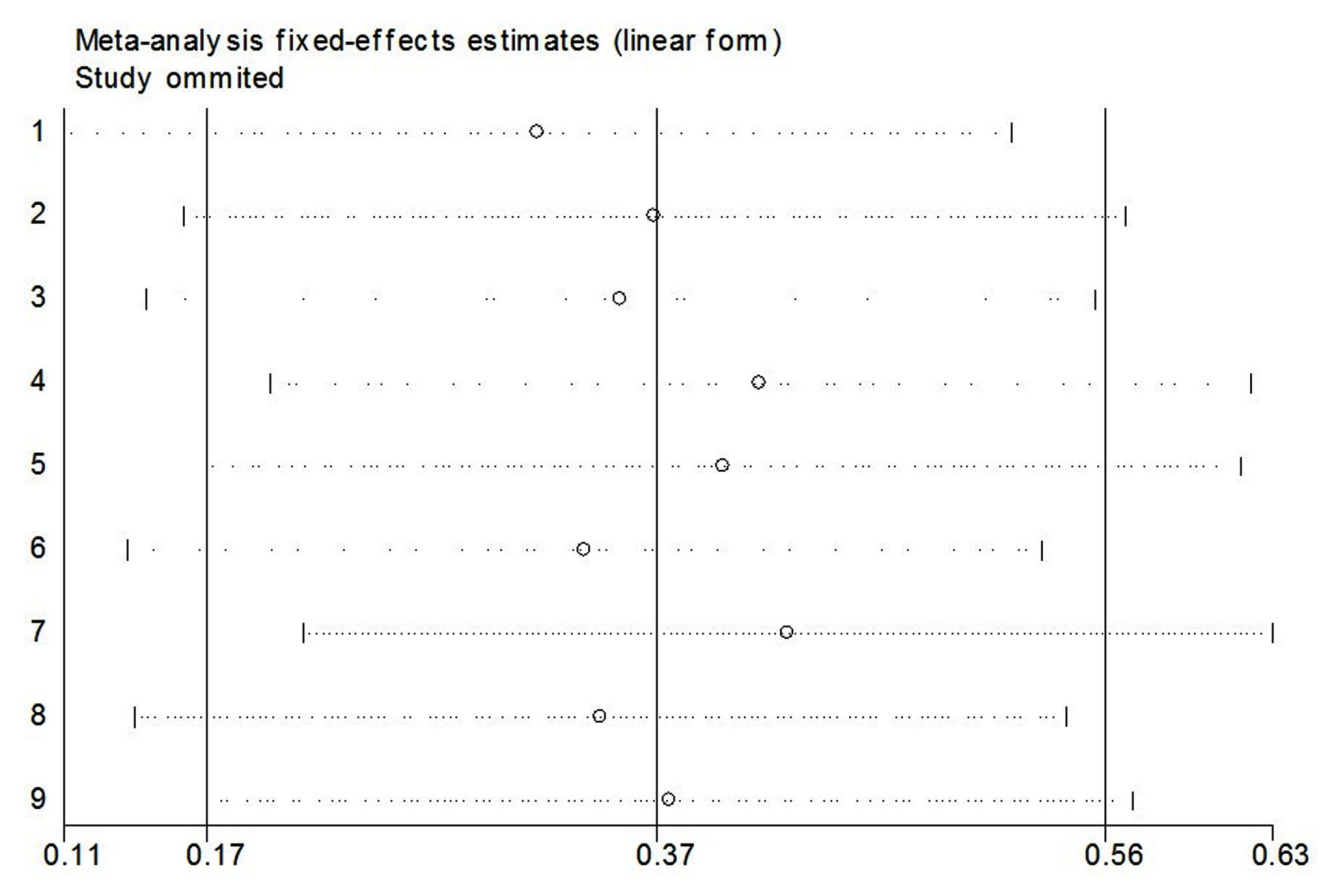
The results of sensitivity analysis from fixed-effects estimates.

Sensitivity analysis was performed by sequentially excluding individual studies, and the summary *ORs* were not materially altered, indicating that our results were statistically robust (Figure 5).

### Publication bias

**Figure 6:**
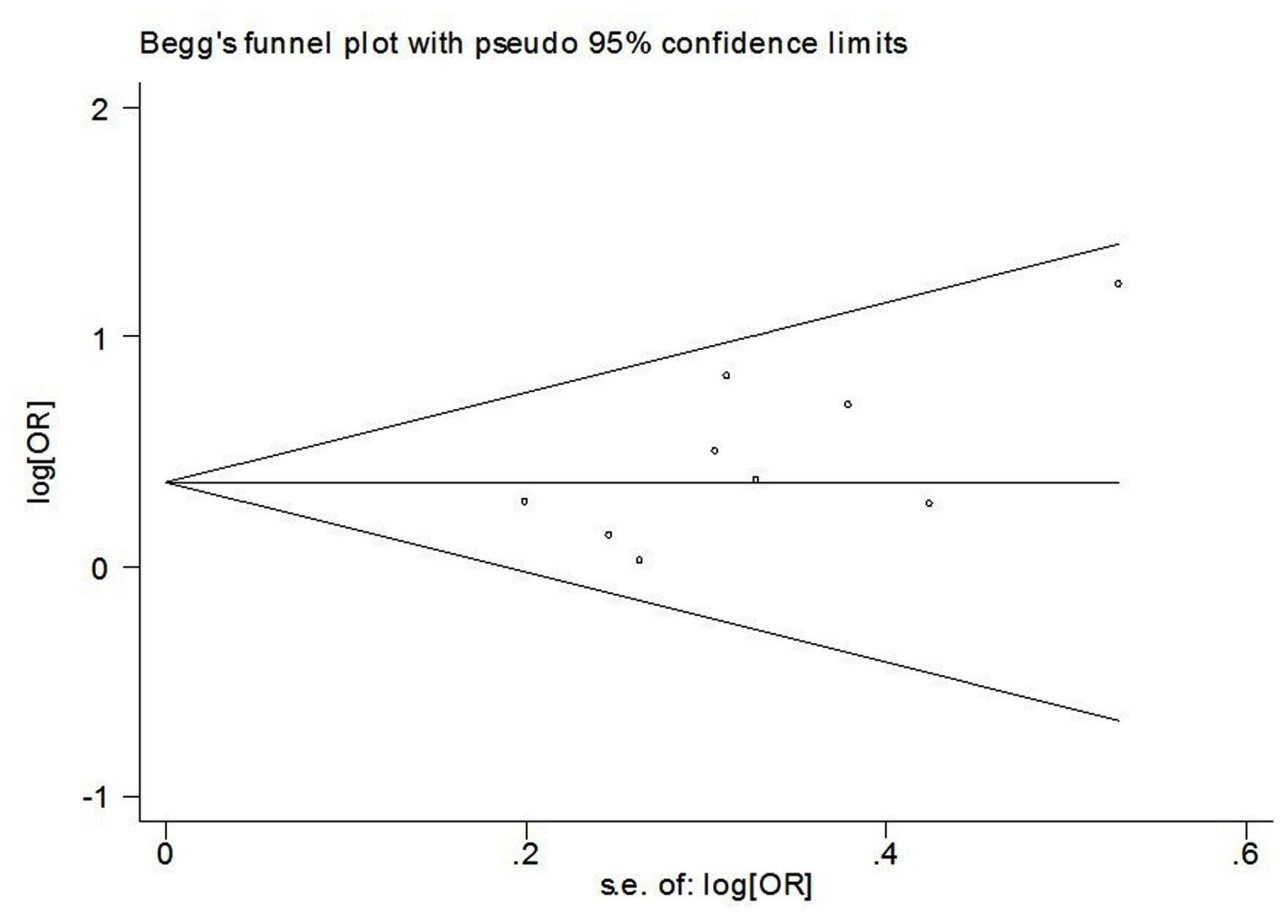
Begg’s funnel plot for publication bias test ( **GA**/**AA** versus GG).

Begg’s funnel plot and Egger’s test were performed to assess the publication bias of the included articles. Figure 6, shows no evidence of obvious asymmetry in the shapes of the funnel plots. The modified Egger linear regression test and Begg’s test indicated that no significant publication bias (*t* =2.23, *P* =0.061; *Z*=1.67, *P*=0.118 for GA/AA vs. GG, Figure 6).

## Discussion

The current meta-analysis suggests a significant relationship between TNF-α-308G/A gene polymorphism and silicosis risk under the dominant genetic model for 1.45(95%CI: 1.20-1.760), but no significant association is found under the recessive genetic model (*OR*=0.77, 95%CI: 0.46-1.29). Thereby, the current study indicates TNF-α-308G allele might increase silicosis risk.

Silicosis as a chronic interstitial lung disease occurs among miners, sand blasters and quarry workers and is manifested as a chronic inflammatory response, which eventually leads to severe pulmonary fibrotic changes [5]. TNF-α is one of the most relevant cytokines to the biological events in silicosis such as inflammation and fibrosis [43,44]. TNF-α is a pleiotropic cytokine that elicits a wide spectrum of physiological and pathogenic events including cell proliferation, differentiation, apoptosis and inflammation. It has been proved to be involved in the fibrosis of many diseases [41-43]. Once the pulmonary tissues are damaged, macrophages would aggregate around the damaged tissues and be activated to release a large amount of TNF-α, which leads to the occurrence of the initial acute alveolar inflammation. On one hand, the TNF-α release induced by macrophages aggregation secretes abundant human transforming growth factor-beta 1 and human monocyte chemoattractant protein-1 and further expands the inflammatory response. On the other hand, TNF-α can also promote the proliferation of fibroblasts and secrete a large amount of collagen. It is suggested that TNF-α is expressed inⅡalveolar epithelial cells but not in normal pulmonary tissues, indicating that the abnormal expression of TNF-α may play an important role during fibrosis [44]. In many studies, serum TNF-α level in the silicosis groups are significantly higher than that in the control groups [45,46].

The genetic contribution of the host plays a key role in determining the susceptibility risk and severity of a disease and the health outcome of a patient [47]. This category includes susceptible genes such as single nucleotide polymorphisms (SNPs), which quantitatively change the expressions production of inflammatory mediators. TNF-α gene -308G/A may be suggested to be responsible for the variation of TNF-a production. Transcription of TNF-α is regulated by upstream 1100 bp promoter region [27]. Generally, G to A substitution at position -308 represents a functional polymorphism which leads to different transcription rates in TNF-α production. The -308 A promoter allele shows stronger transcriptional activity than the wild type [48]. Consequently, the allele A of TNF-308 polymorphism may lead to increased expression of the TNF-α gene. And individual differences of TNF-α from monocyte are inferred to be, at least partly, dependent on genetic polymorphism.

The present meta-analysis of nine studies provides more comprehensive analysis on the relationship between TNF-α-308A/G gene polymorphism and susceptibility to silicosis. The genotype GA/AA vs. GG is associated with the silicosis risk, whereas the genotypes (AA vs. GG; AA vs. GA/GG) seem to have no significant association with the silicosis risk in the total population. These findings show that TNF-α-308G/A polymorphism might help to explain the individual differences in the susceptibility to silicosis. Considering the effect of genetic background on the results, we also performed subgroup analysis with respect to ethnicity. Because of few studies on non-Asians, the subgroup analysis was carried out for Asians. The results show that the Chinese people with the genotype GA/AA vs. GG may be more susceptible to silicosis. We found no significant association for AA versus GG model and AA versus GA/GG model, which might be attributed to the low statistical power due to the low AA genotype frequency. Meanwhile, no significance was found in non-Asians for the genotype GA/AA vs. GG; the possible reasons of such a result may be the differences in the genetic backgrounds and living environments. In addition, only two included studies were non-Asians, which made the stratified analysis not so reliable. Therefore the result should be interpreted with caution, and additional studies with further large-scale case-control ones are needed to validate the result. However, according to the power analysis, our study had greater than 80% to detect the effects of TNF-α polymorphism on silicosis, assuming an *OR* of 1.45. This meta-analysis at least provides new clues to the ethnic differences.

The overall comparisons did not show heterogeneity or publication bias, indicating that the results were statistically robust. However, some limitations from the following aspects should be addressed. First, a disease is largely affected by the interaction between genotypes and environment. People from different places have specific work environments, and genetic characteristics may be different among races, which should be considered in the study. Moreover, silicosis usually has a long developing stage that can be affected by a number of factors such as age, sex, working tenure, and many of them were not included in this study because of the lack of original data, which cause some bias. Second, larger sample sizes are necessary in future studies to eliminate the interference factors of genetic risk contributed by the *Q* variants of model effects, particularly when they have a severe effect in a recessive model. Finally, our result was based on unadjusted estimates; therefore, more precise analysis should be performed if individual data were attainable, which would allow for the adjustment by other factors, including age, family history and environmental factors.

Despite those limitations, this systematic review of the association of TNF-α-308G/A gene polymorphism with silicosis risk is statistically more convincing than any single study. This meta-analysis based on a large sample size strongly gives a strong conclusion that the -308G/A polymorphism might lead to an increased risk of silicosis susceptibility, especially for Asians. However, in order to better to estimate the association of other variables with the susceptibility to silicosis, it is necessary to carry out further researches with a larger number of worldwide studies in standardized and unbiased ways.

## Supporting Information

Checklist S1 **Prisma checklist.**
(DOC)
